# Combining Spinach-tagged RNA and gene localization to image gene expression in live yeast

**DOI:** 10.1038/ncomms9882

**Published:** 2015-11-19

**Authors:** David Guet, Laura T. Burns, Suman Maji, Jérôme Boulanger, Pascal Hersen, Susan R. Wente, Jean Salamero, Catherine Dargemont

**Affiliations:** 1 Univ Paris Diderot, Sorbonne Paris Cité, INSERM UMR944, CNRS UMR7212, Equipe labellisée Ligue contre le cancer, Hôpital St Louis, 1 Avenue Claude Vellefaux, 75475 Paris Cedex 10, 75475, France; 2 Department of Cell and Developmental Biology, Vanderbilt University School of Medicine, 205 Kirkland Hall, Nashville, Tennessee 37232-8240, USA; 3 Team-Space Time Imaging of Endomembranes and Organelles Dynamics, UMR144 CNRS, Univ Pierre et Marie Curie, Institut Curie, 12 rue Lhomond, Paris 75005, France; 4 Univ Paris Diderot, Sorbonne Paris Cité, CNRS UMR7057, Laboratoire Matière et Systèmes Complexes, 10 rue Alice Domon et Léonie Duquet, Paris 75013, France; 5 PICT-IBiSA Imaging Core Facility, Institut Curie, 12 rue Lhomond, Paris 75005, France

## Abstract

Although many factors required for the formation of export-competent mRNPs have been described, an integrative view of the spatiotemporal coordinated cascade leading mRNPs from their site of transcription to their site of nuclear exit, at a single cell level, is still partially missing due to technological limitations. Here we report that the RNA Spinach aptamer is a powerful tool for mRNA imaging in live *S. cerevisiae* with high spatial-temporal resolution and no perturbation of the mRNA biogenesis properties. Dedicated image processing workflows are developed to allow detection of very low abundance of transcripts, accurate quantitative dynamic studies, as well as to provide a localization precision close to 100 nm at consistent time scales. Combining these approaches has provided a state-of-the-art analysis of the osmotic shock response in live yeast by localizing induced transcription factors, target gene loci and corresponding transcripts.

## Abstract (web summary)

Measuring single-cell mRNA dynamics is critical to understand gene expression. Here, using RNA Spinach technique to detect very low abundant mRNAs, Guet *et al*. report an analysis of the osmotic shock response in live yeast by localizing induced transcription factors, target gene loci and corresponding transcripts.

## Graphical abstract

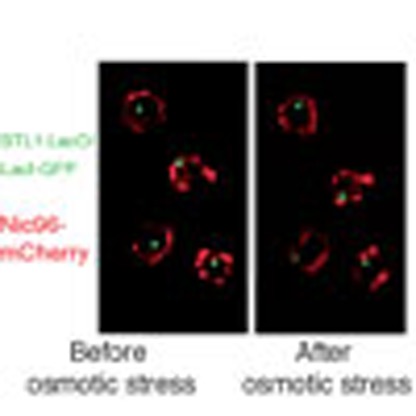

Thirty years ago, Blobel^1^ suggested that ‘the nuclear pore complexes (NPC) are envisioned to serve as gene-gating organelles capable on interacting specifically with expanded (transcribable) portions of the genome'. This ‘platform concept' would satisfy spatial coordination constraints by setting messenger RNA biogenesis machineries in the vicinity of transcribing genes and locating transcribed mRNA close to the nuclear exit sites.

In agreement with this hypothesis, recent studies in yeast *Saccharomyces cerevisiae* highlight a role for the NPC in promoting and orchestrating gene expression by confining transcription, mRNA processing, quality control and nuclear transport processes in a defined nuclear microenvironment^2^,^3^,^4^. Specific *cis*-acting sequences and promoters, transcriptional activators, the coactivator SAGA, 3′-untranslated regions (UTRs), the exosome, mRNA export factors and some NPC components (Nups) have all been implicated in ‘gene gating'^3^. Localization of gene loci is usually analysed using insertion of LacO (or TetO) repeats downstream of the gene of interest in cells expressing GFP–LacI (or TetR) repressor and a fluorescently tagged Nup^5^. This approach allows monitoring of gene movements towards the nuclear periphery on transcription induction, but the low-resolution microscopy approaches used to date prevent a precise localization of gene loci within the nucleus of live yeast cells.

Measuring the single-cell dynamics of mRNA molecules is also critical to understanding the mechanisms regulating gene expression. Fluorescent *in situ* hybridization (RNA FISH) is a method of choice to detect transcripts *in situ*. In recent times, the FISH technique was adapted to allow quantification and localization of RNAs at single-molecule resolution in yeast cells^6^. However, the methods required for hybridization necessitate the use of fixed material and as such, visualizing the site of transcription by FISH is far from precise, in particular in yeast nuclei where the diameter is in a micrometre range. In addition, the timescale of mRNA trafficking from the site of transcription to the NPC, and ultimately to the cytoplasm, is not compatible with accurate FISH analysis, unless this process is stalled by using appropriate mutants.

To date, localization of endogenous mRNAs in living cells has mostly relied on the insertion of the RNA-binding sites for the MS2 or *Pseudomonas* phage PP7 coat proteins between the coding region and the 3′-UTR of the gene of interest. Co-expression of a respective coat protein fusion with tandem green fluorescent proteins (GFPs) then allows analysing mRNA localization by classical fluorescence microscopy. However, this method has inherent limitations. The high number of MS2- or PP7-binding sites, as well as the tandem GFPs used to increase the signal, result in constant high background and might affect the correct coupling between 3′-end processing and trafficking, alter the formation of an export-competent mRNP and create alterations in the resolution of mRNA localization^7^,^8^,^9^. Split fluorescent proteins have recently been used in an attempt to overcome the constant background inherent to these approaches^10^. However, all MS2 or PP7-based approaches display common photobleaching and consecutive phototoxic effects that preclude, at least in yeast cells, dense regime of acquisition or long-term imaging.

Here we report an alternative approach using the Spinach aptamer to localize mRNA in living yeast that has minimal photobleaching effect and low fluorescent background, as well as marginal perturbation of mRNA biogenesis, to allow the study of export-competent mRNP formation. This is completed by imaging workflows that combine multi-points confocal microscopy, a time adaptive denoising algorithm and deconvolution, leading to a localization precision close to 100 nm and giving access to various time scales. Finally, these approaches are challenged, to provide an integrative view of the yeast cell response to osmotic shock by localizing induced transcription factors, target gene loci and corresponding transcripts in three dimension (3D).

## Results

### Spinach aptamer as a tool for mRNA imaging in live yeast

A recently published study described a short 80-nucleotide-long RNA aptamer (Spinach) that emits green fluorescence comparable in brightness to enhanced GFP on binding with 3,5-difluoro-4-hydroxybenzylidene imidazolinone (DFHBI)^11^,^12^. To test whether this probe was adaptable for localizing RNA in live yeast cells, we developed genetic tools to insert the Spinach sequence between the coding region and the 3′-UTR of any gene of interest in *S. cerevisiae* genome. Specifically, we adapted the strategy used for integrating binding sites for the RNA-binding MS2 coat protein^13^. In this, the selection marker is flanked by loxP sites, to allow its excision on Cre recombinase expression (Supplementary Fig. 1a). By doing so, perturbations of the tagged mRNA properties (expression, localization and trafficking) due to the insertion of Spinach are probably minimized.

**Figure 1:**
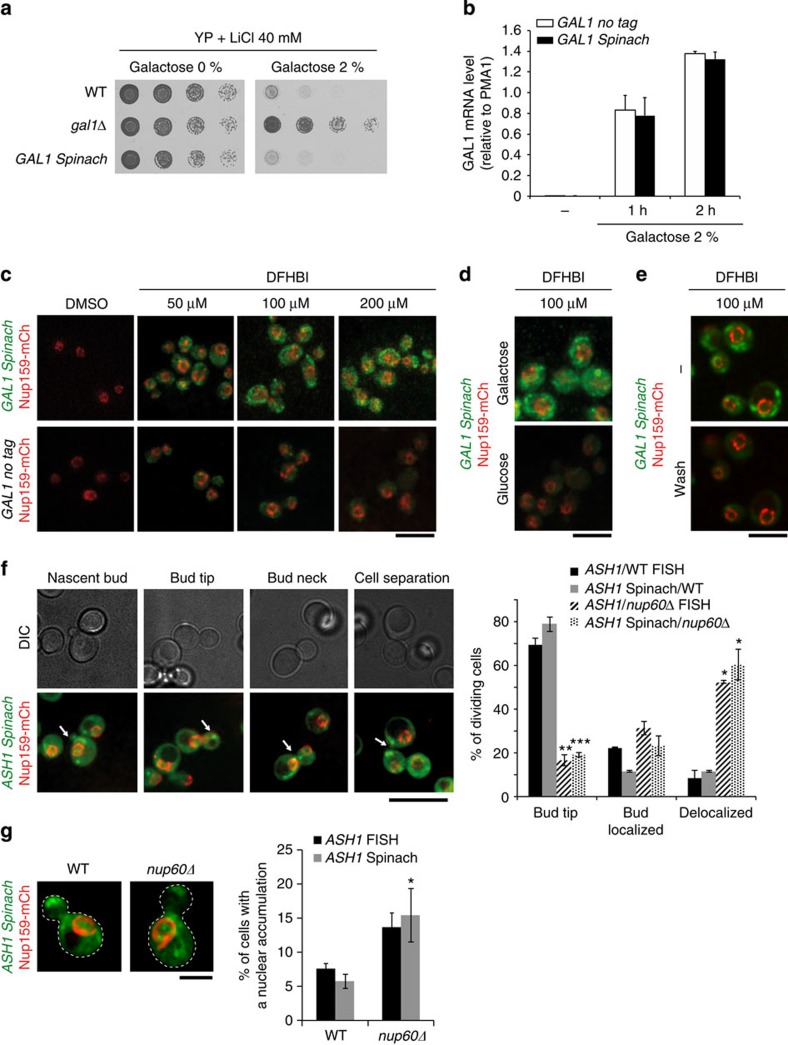
Use of Spinach RNA aptamer to monitor localization of mRNAs in *S. cerevisiae*. (**a**) Spinach tagging does not alter the function of tagged *GAL1* transcript. Fivefold serial dilutions of strains grown on the indicated media. (**b**) Expression of *GAL1 Spinach* transcripts. Expression of tagged and untagged *GAL1* transcripts were analysed by RT–qPCR in cells grown overnight in raffinose and shifted to galactose for 1 or 2 h. Expression analysis was performed in three replicates. (**c**–**e**) Localization of *GAL1 Spinach* transcripts. After overnight culture in raffinose at 30 °C, cells were grown for 2 h in galactose (**c**) or in glucose (**d**) and incubated with indicated concentration of DFHBI. (**e**) Cells grown in galactose for 2 h and incubated with 100 μΜ DFHBI were washed and observed just after DFHBI removal. Scale bar, 10 μm. (**f**) Localization of *ASH1* mRNAs scored by Spinach tagging or FISH. Examples of *ASH1 Spinach* transcripts localization at different stages of the cell cycle. *ASH1* transcripts are visible as dots (arrows) in the nucleus or the cytoplasm of the cells and Nup159–mCherry reveals the nuclear periphery (left panel). Histogram showing the distribution of *ASH1* transcripts analysed using Spinach tagging or FISH in wt or *nup60*Δ dividing cells (right panel, Spinach tagging analysis was performed in three replicates with *n*=68 for wt and *n*=58 for *nup60*Δ cells; FISH analysis was performed in two replicates with *n*=67 for wt and *n*=92 for *nup60*Δ cells). Scale bar, 10 μm. (**g**) Deletion of *NUP60* promotes nuclear retention of *ASH1* transcripts in the mother cell. Nuclear accumulation was analysed by Spinach tagging (left panel), and quantified and compared with FISH analysis (right panel). Spinach tagging analysis was performed in three replicates with *n*=356 for wt and *n*=369 for *nup60*Δ cells; FISH analysis was performed in two replicates with *n*=222 for wt and *n*=400 for *nup60*Δ cells. Scale bar, 2 μm. Error bars show s.d. Results have been compared using a Student's *t*-test, ***P<0.001,**0.001<*P*<0.01, *0.01<*P*<0.05. Image acquisition of Spinach-tagged transcripts was done with exposure set at 200 ms with 30% of a 50 mW laser. Saturation of the signal was observed for exposure set above 300 ms.

To validate this technology, the Spinach aptamer was first introduced in the galactose-inducible *GAL1* gene and the *ASH1* gene encoding constitutive polarized RNAs. To test whether the Spinach aptamer altered the function of tagged *GAL1* transcript, cell viability was analysed on addition of galactose and lithium. Deletion of *GAL1* indeed prevents the galactose toxicity in the presence of lithium^14^. However, insertion of Spinach did not confer any growth rescue in these experimental conditions (Fig. 1a). In addition, the Spinach tag did not affect gene expression as attested by reverse transcriptase–quantitative PCR (RT–qPCR) measurements (Fig. 1b). These results show that the Spinach aptamer did not significantly modify the function and induction of tagged *GAL1* transcript.

Owing to the very low abundance of RNA polymerase II-derived mRNAs in yeast, a weak Spinach-tagged RNA signal combined with a low signal-to-noise ratio were anticipated. To overcome this limitation, spinning-disk confocal microscopy acquisition was combined with a space- and time-adaptive denoising algorithm that exploits redundancy in the image sequence^15^,^16^. The expression of inducible *GAL1 Spinach* transcripts was detected in a timescale consistent with data obtained by RT–qPCR. Importantly, the fluorescence signal was dependent on the Spinach aptamer and was induced by the activity of the *GAL1* gene (galactose) and on addition of DFHBI (Fig. 1c,d). Remarkably, the fluorescent signal decreased within a few seconds after DFHBI removal, thereby confirming the rapid exchange between the Spinach aptamer and DFHBI (Fig. 1e)^11^, a clear asset of the system in limiting the photobleaching and its cytotoxic effects.

Analysis of *ASH1 Spinach* transcripts indicated that *ASH1* mRNA was primarily localized at the bud tip in small and medium budded cells prior migrating to the bud neck in late G2–M phase (Fig. 1f). This was comparable to previous reports of MS2-tagged *ASH1* (ref. 17) or *ASH1* transcripts localized by FISH^18^ (Fig. 1f and Supplementary Fig. 1b) and indicates that the cell-cycle-dependent localization of this transcript was not altered by the Spinach aptamer insertion. The proper intracellular trafficking of endogenous *ASH1* transcripts, nuclear export and cytoplasmic localization depends on specific proteins of the NPC, and in particular Nup60 in the nuclear basket NPC substructure^18^ (Fig. 1f,g). Similarly, we found that Spinach-tagged *ASH1* mRNAs were partially retained in the nucleus of *nup60Δ* cells and were mislocalized in the cytoplasm (Fig. 1f,g).

Together these findings indicate that introduction of the Spinach aptamer between the coding region and the 3′-UTR of the gene of interest allows detection of specific transcripts in live yeast without affecting their expression or subcellular trafficking. In addition, the Spinach approach overcomes two limitations of the MS2- or PP7-based approaches, the constant fluorescent background due to the expression level of the GFP-tagged protein and photobleaching effects.

### Time-lapse analysis of *STL1* expression on osmotic stress

To further assess the potential of the Spinach technology for dynamic studies, in particular during mRNA biogenesis, we precisely analysed *STL1*, a gene transiently induced on osmotic shock. According to RT–qPCR and FISH analysis, *STL1* mRNA expression peaked at 7–10 min after induction with a nuclear dot appearing after 5 min in 0.4 M NaCl followed by a cytoplasmic punctuate staining (Supplementary Fig. 2).

**Figure 2:**
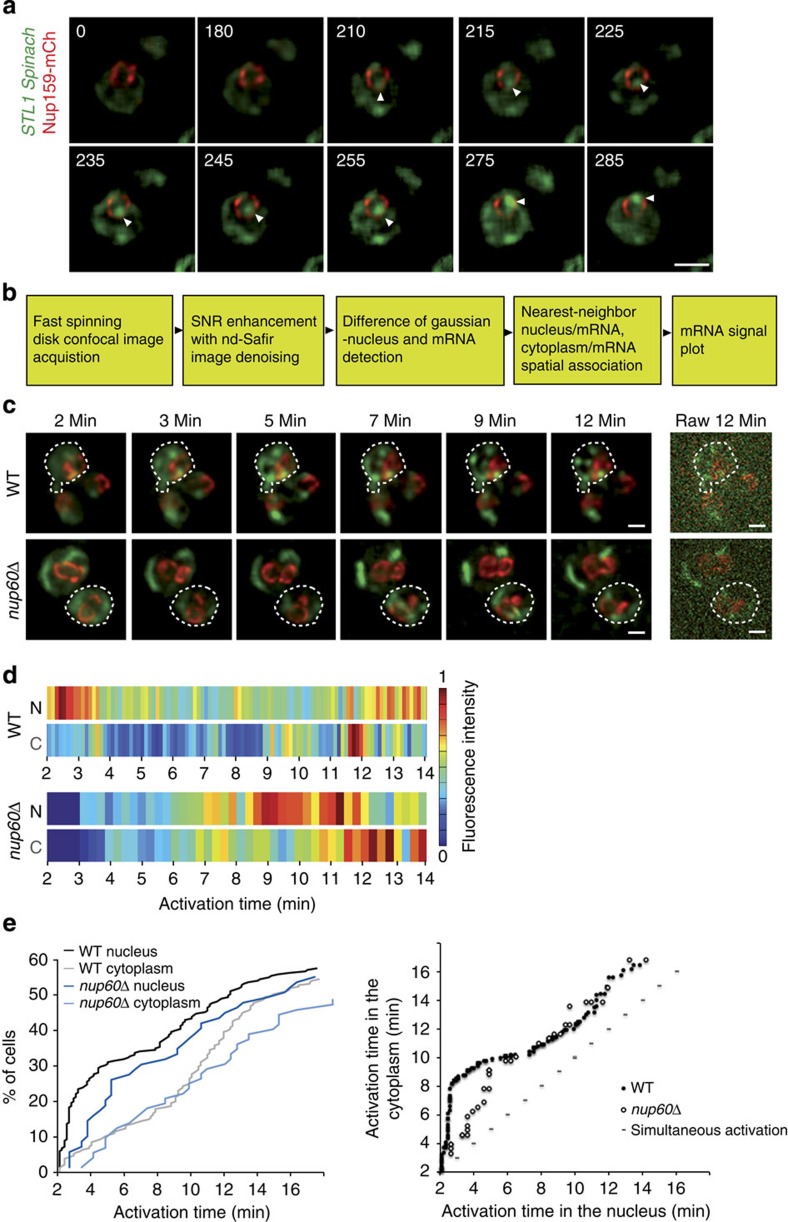
Precise spatiotemporal analysis of *STL1 Spinach* expression. (**a**) Fast time-lapse movie of *STL1* activation. *STL1 Spinach* signal induced on treatment with 0.4 M NaCl was registered 2 min after the induction, to avoid side effects of cell shrinkage. Images were acquired by spinning-disk microscopy on a single plane with one frame per second for 5 min. Nuclear transcripts appeared as a nuclear dot indicated by a white arrow. The nuclear periphery was visualized with Nup159–mCherry. Scale bar, 2 μm. See Supplementary Movie 1. (**b**) Worflow scheme developed to image, detect and analyse Spinach-tagged RNA in live yeast. (**c**) Induction of *STL1* transcript in wt and *nup60Δ* living cells. *STL1 Spinach* signal induced as in **a** was recorded 2 min after induction with one Z stack of 11 frames per 5 s for wt and one Z-stack of 14 frames per 20 s for *nup60Δ* cells during 12 min. Images correspond to maximum projections of denoised spinning-disk Z-stack images (11 and 6 frames for wt and *nup60Δ*) at precise time points. Unprocessed Z-stack projections corresponding to the last time point are shown on the right (raw). Scale bar, 1 μm; see Supplementary Movies 2 and 3. (**d**) Heat maps. Changes of fluorescence intensity of *STL1-*Spinach dots from cells shown in **c** were measured in 3D, inside the nucleus (N) and in the cytoplasm (C). The colour bar indicates fluorescence intensity, with dark blue for the absence of fluorescence and red indicating maximum fluorescence presence. (**e**) Kinetics of *STL1* activation. The time of first appearance of *STL1* transcripts, in the nucleus (black and dark blue for wt and *nup60Δ*cells) and in the cytoplasm (grey and light blue for wt and *nup60Δ* cells), was defined for each single cell from the heat maps (*n*=235 wt cells; *n*=72 *nup60Δ* cells). The cumulative distribution, corresponding to the % of activated cells that display the first detection of a Spinach signal at a given time point was plotted as a function of activation time (left panel). The first occurrence of the Spinach signal in the cytoplasm was also plotted as a function of the first occurrence of the nuclear signal in each single cell (right panel). Each dot corresponds to a single cell. The theoretical line corresponding to a simultaneous appearance of the signal in the nucleus and cytoplasm is represented by the dashed line.

To monitor transcript induction in living cells, cells co-expressing *STL1 Spinach* mRNA and the NPC protein Nup159–mCherry were imaged on one medial single frame using spinning-disk confocal microscopy at a relatively high frame rate (1 f s^−1^) for 5 min (Supplementary Movie 1). The nuclear dot dynamics were well appreciated in some cells (Fig. 2a). Unfortunately, the frequent vanishing of the small dot signal in out-of-focus planes in this condition precluded relevant statistical analysis of its dynamic behaviour, in a sufficient number of cells. To accurately monitor transcript induction and statistically analyse its dynamic behaviour, a dedicated imaging workflow was designed (Fig. 2b). First, 3D Z-stacks of 11 planes spaced by 0.3 μm were acquired at 1 frame per 150 ms every 5 s for 12 min using spinning-disk confocal microscopy. The continuous exchange between free DFHBI and Spinach minimized potential photobleaching problems of the Spinach-DFHBI signal during acquisition. However, the low copy number of transcripts led to a low signal-to-noise ratio for Spinach-DHFBI. In addition, the use of excess light illumination, which would be necessary to assess the positioning of the NPCs within the 1,580 frames, would induce photobleaching on Nup159–mCherry and an undesired phototoxicity as previously measured^15^. All these constraints were overcome through the integration of image denoising post processing as a second step of the workflow. It allowed the Spinach signal and Nup159–mCherry to be recorded at the same rate and at very low signal-to-noise ratio with minimal phototoxicity, even at relatively dense regime of acquisition over a long time of acquisition (Fig. 2c), Transcripts and NPC signals were automatically detected using a difference of Gaussian (DoG) filter and a threshold defined by a *P*-value. NPC signal was then traced across the time-lapse sequence of 3D stacks and the fluorescent intensity corresponding to *STL1 Spinach* mRNAs was measured over time both in the nucleus and in the cytoplasm. The data were represented using intensity heat maps corresponding to each cell and illustrated the cell-to-cell variability of the STL1 expression^5^,^19^ (Fig. 2d and Supplementary Fig. 3).

The appearance of the *STL1* mRNA in the nucleus and the cytoplasm was calculated for each single cell over time and the time of first appearance of *STL1* transcripts was defined in the nucleus and corresponding cytoplasm of each cell. The cumulative distribution (corresponding to the % of activated cells that display the first occurrence of a Spinach signal at a given time point) was plotted as a function of activation time, to compare the kinetics of STL1 mRNA induction and expression in both cell compartments on the whole-cell population (Fig. 2e left panel). Alternatively, the first occurrence of the Spinach signal in the cytoplasm was plotted as a function of the first occurrence of the nuclear signal in each single cell (Fig. 2e right panel). Using these quantitative approaches, we first observed that only 60% of wild-type (wt) cells expressed *STL1 Spinach* transcript on osmotic shock (Fig. 2e left panel) as previously referred to stochastic gene activation and inhibitory role of casein kinase 2 (refs 5, 19, 20). *STL1 Spinach* mRNA was detected as a nuclear dot close to the nuclear periphery in 50% of induced cells within 3.5 min and as cytoplasmic particles within 6 min after the osmotic shock (Fig. 2c–e and Supplementary Movie 2). In addition, the first occurrence of the Spinach signal always appeared in the nucleus and then in the cytoplasm, thus supporting the validity of our approach and analysis (Fig. 2e right panel). Deletion of *NUP60* gene not only alters nuclear export and cytoplasmic localization of polarized transcripts such as *ASH1* but also affects transcription of osmotic stress-responsive genes including *STL1* (ref. 21). In agreement, we found that the absence of Nup60 led to a 3-min delay in *STL1* mRNA expression with an expression peak varying from 10 to 20 min (Supplementary Fig. 2a) that resulted in a 3-min delay of transcript nuclear export using both Spinach aptamer-based bioimaging (Fig. 2c–e and Supplementary Movie 3) and FISH analysis (Supplementary Fig. 2c). Reliably, production of the STL1 protein was detected at later time points after osmotic stress in *nup60Δ* cells (Supplementary Fig. 2b). However, the correlation between time of appearance in the cytoplasm and nucleus in wt and mutant cells indicates that once transcripts are produced in the nucleus, they are exported to the cytoplasm with the same kinetics, at least when produced after 5 min of NaCl treatment (Fig. 2e right panel). Together, these data validate the use of Spinach technology for dynamic studies of mRNA biogenesis in live yeast.

These results highlight the accuracy of our methodological approach to carefully analyse kinetics of mRNA expression and localization at the single-cell level and thus allow us to quantify mRNA biogenesis or export defects with an appropriate timescale and with a limited risk of phototoxicity.

### Workflow for localization of *STL1* gene locus in live yeast

**Figure 3:**
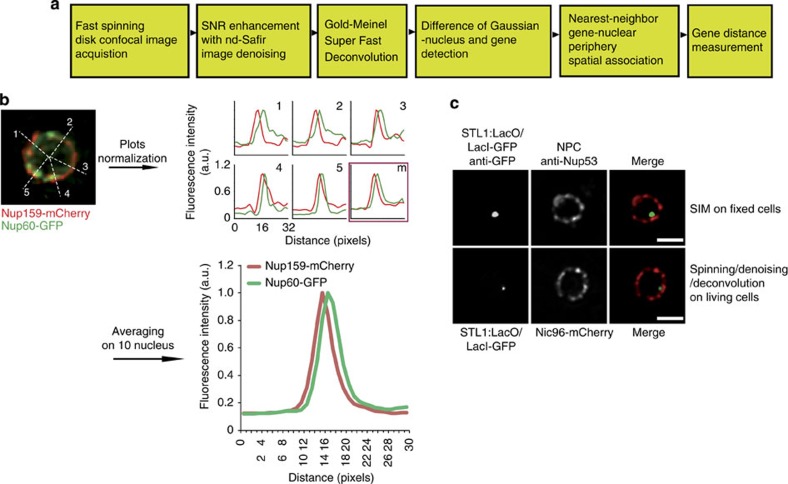
Development of an imaging workflow to localize gene loci in live yeast. (**a**) Imaging workflow scheme. (**b**) Imaging workflow differentiates nuclear and cytoplasmic sides of the NPC. The nuclear pore complex was visualized on cells expressing Nup159–mCherry on the cytoplasmic side and Nup60–GFP on the nucleoplasmic side. Z-stack spinning-disk images (300 ms per frame, both channel) were denoised and deconvolved. Fluorescence intensity profiles were measured centrifugally in both fluorescent channels, along the indicated line axes shown on the left (red, Nup159–mCherry; green, Nup60–GFP). Centroid distances between fluorescent signals were determined for each line axis and corresponding plots (1 to 5), as well as an average plot are shown for the indicated nucleus on the right. Results pooled from ten different nuclei shown in the lower part of the figure indicate a localization accuracy with 1 pixel precision (64.5 nm) between signals of both Nups. (**c**) Comparison of structural illumination microscopy (SIM) and spinning-disk workflow. For SIM, the *STL1* gene locus was localized on fixed cells using anti-GFP targeting of GFP–LacI linked to a 128-LacO array inserted into the 3′-UTR of *STL1* and anti-Nup53 antibodies (red). In spinning-disk, the *STL1* gene locus was localized in living cells by expression and targeting GFP–LacI and the NPC by Nic96–mCherry. After acquisition in spinning disk, images were denoised and deconvolved. Images correspond to 1 medial frame, pixel size 64.5 nm. Scale bar 1 μm.

To determine whether the dynamic mRNA nuclear dot corresponds to the site of transcription, we next adapted our integrated imaging approach to precisely localize the corresponding gene in live yeast. The position of the *STL1* gene compared with the NPC was analysed by insertion of 128-Lac0 repeats downstream in the 3′-UTR of *STL1* in cells expressing GFP–LacI repressor and Nic96–mCherry^22^. The challenge was to accurately improve both the signal-to-noise ratio and the localization precision, while keeping imaging compatible with acquisition in live cells. We thus complemented the imaging suite described before, based on spinning-disk confocal microscopy acquisition combined with adaptative denoising, by adding a deconvolution step based on point spread function (PSF) measurement and the Gold–Meinel algorithm^23^ (Fig. 3a). This deconvolution method showed a good robustness to the inaccuracy of the PSF and we previously demonstrated that its performance is even improved if denoising is applied beforehand^16^. Gene locus and NPC signals were then automatically detected using a DoG filter (see methods; Fig. 3a).

To test the localization precision accuracy of this imaging workflow, cells expressing both Nup60–GFP and Nup159–mcherry were analysed. Fluorescence intensity profiles along five radial line axes per nucleus were measured and centroid distances between fluorescent signals determined and plotted (Fig. 3b). Averaged results from ten different nuclei indicated that this approach allows us to distinguish both Nups, known to be distant by about 50 nm^24^, with a localization precision of 1 pixel (64.5 nm). This simple imaging workflow provided consistent results with what could be achieved by high-resolution structured illumination microscopy reconstruction approaches^25^,^26^ within a timescale (1 frame per 150 ms) compatible with live yeast imaging.

We finally validated the use of this approach for gene positioning by comparing the localization of the *STL1* gene locus against the distribution of the NPC, in experiments using either 3D structure illumination microscopy on fixed cells (1 frame per 2.3 s, 15 frames, total time of acquisition: 79.3 s) and the above imaging workflow in live yeast (1 frame per 150 ms, 11 frames, total time of acquisition: 3.5 s; Fig. 3c).

### Localization of the *STL1* gene locus on osmotic stress

**Figure 4:**
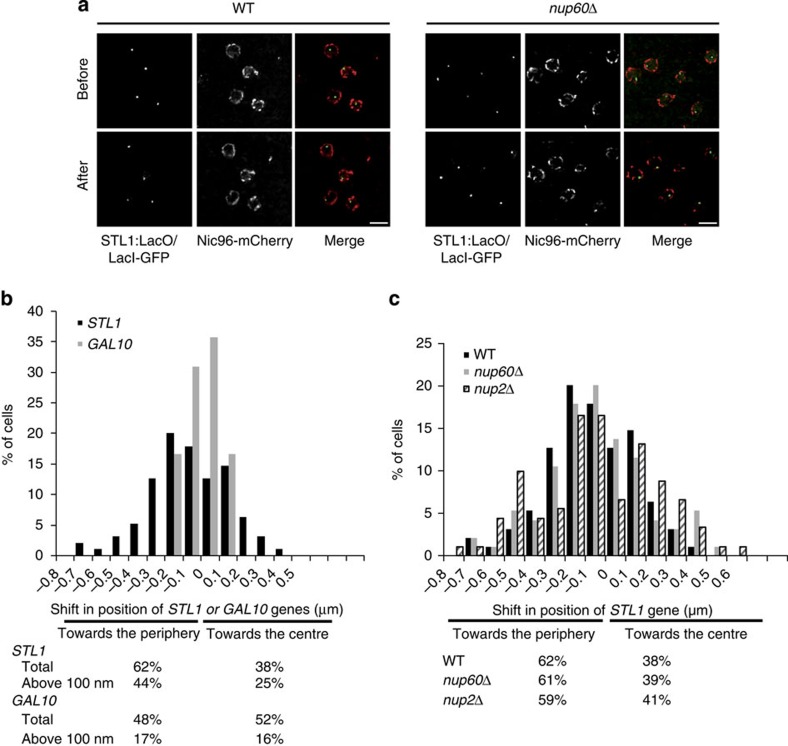
Localization of *STL1* gene locus in live yeast. (**a**) Imaging *STL1* gene positioning on activation. Cells were imaged for Nic96–mCherry and GFP–LacI before and after 10 min activation with NaCl 0.4 M and analysed using the imaging workflow validated in Fig. 3. Scale bar, 2 μm. (**b**) *STL1* and *GAL10* gene positioning on activation in wt cells. The shift in position of the gene was calculated in 3D on *n*=95 cells for *STL1* and *n*=42 for *GAL10* recorded as in **a**. A negative shift corresponds to the gene moving close to the nuclear periphery, whereas a positive shift describes movement towards the centre of the nucleus. Total % of cells presenting a shift of *STL1* or *GAL10* gene loci or % of cells presenting a shift above 100 nm of *STL1* or *GAL10* gene loci towards the periphery or the centre is indicated. (**c**) *STL1* gene positioning on *NUP60* and *NUP2* deletion. The shift in position of the gene was calculated in 3D on *n*=95 wt cells, *n*=95 *for nup60Δ* cells and *n*=91 for *nup2Δ* cells recorded as in **a**. Total % of cells presenting a shift of *STL1* gene locus towards the periphery or the centre is indicated for the different strains.

We next analysed cells expressing Lac0-tagged *STL1*, Nic96–mCherry and GFP–LacI repressor before and after osmotic shock (Fig. 4a). The shape of the nucleus, defined by Nic96–mCherry, and *STL1* gene locus were detected using DoG filters. The Euclidean distance between the gene centroid and the nearest pixel of the nuclear periphery, taking into account the anisotropy of the voxel (64 × 64 × 300 nm) for the acquired volume, was calculated before and after osmotic stress. The difference between the final and initial distance, when negative, indicated the movement of a gene locus towards the nuclear periphery. On activation, the *STL1* gene relocated to the periphery of 62% wt cells (with a shift above 100 nm towards the periphery in 44% of cells) browsing an averaged distance of 305 nm (Fig. 4b). These results were consistent with those obtained using quantification of peripheral nuclear localization in two dimensions (Supplementary Fig. 4a; 22) and with the percentage of cells that transcribe *STL1* mRNA in these activation conditions (Fig. 2). Similar results were found with *CTT1*, another osmostress-inducible gene (Supplementary Fig. 4b). In contrast, the localization of the osmostress non-inducible *GAL10* gene was unchanged on osmotic shock with a shift above 100 nm towards the periphery in only 17% of cells and a mean shift of 75 nm towards the periphery or the centre of the nucleus (Fig. 4b). This indicates that genes induced by osmotic shock specifically relocate towards the nuclear periphery on induction.

To further investigate mechanisms and consequences of *STL1* gene relocation on osmostress, localization of this gene locus was analysed before and after osmotic shock in cells deleted for either *NUP60* or *NUP2*, another component of the NPC nuclear basket (Fig. 4a,c and Table 1). Deletion of *NUP60* did not affect the repositioning of *STL1* or *CTT1* gene loci towards the nuclear periphery on osmotic stress (Fig. 4c,d and Supplementary Fig. 4c). In contrast, deletion of *NUP2* led to *STL1* but not *CTT1* gene movements of high amplitude, both towards the periphery and the centre of the nucleus but without affecting the overall redistribution of the *STL1* gene towards the periphery (Fig. 4c,d and Supplementary Fig. 4c). In addition, the position of the *STL1* gene locus before the osmotic stress was more distant from the nuclear periphery (Table 1). These results suggest that specific components of the nuclear basket, such as Nup2, would constrain motility of some genes and might facilitate the residency of induced genes at the nuclear periphery. It is important to note that both Nup60 and Nup2 are required for correct *STL1* mRNA expression and nuclear export (Fig. 2 and ref. 21), thus indicating that the active gene repositioning at the nuclear periphery is not sufficient to control their kinetics of expression.

Our results also allowed measurement of an average distance of 350 nm between the induced *STL1* gene and nuclear periphery (Table 1). In addition, with the improvement of localization precision provided by our imaging approach, an apparent overlap between the induced *STL1* gene locus and the NPC was restricted to only 7% of the wt cells after 10 min induction (*n*=300). Together these data suggest that, although the induced *STL1* gene relocates towards the nuclear periphery on induction, the *STL1* gene locus is possibly only rarely localized at the nuclear envelope. Alternatively, its association with the nuclear periphery, and by interference with the NPC, is very transient, making a stable tethering process unlikely. In contrast, the coincidence of the galactose-induced *GAL10* gene locus signal with the nuclear periphery was found in 24% of cells (not shown), suggesting that the time of residency of an induced gene at the yeast nuclear periphery might depend on the particular gene and its specific interaction with the peripheral microenvironment.

### Localization of stress-induced Hot1 with *STL1* gene and mRNA

**Figure 5:**
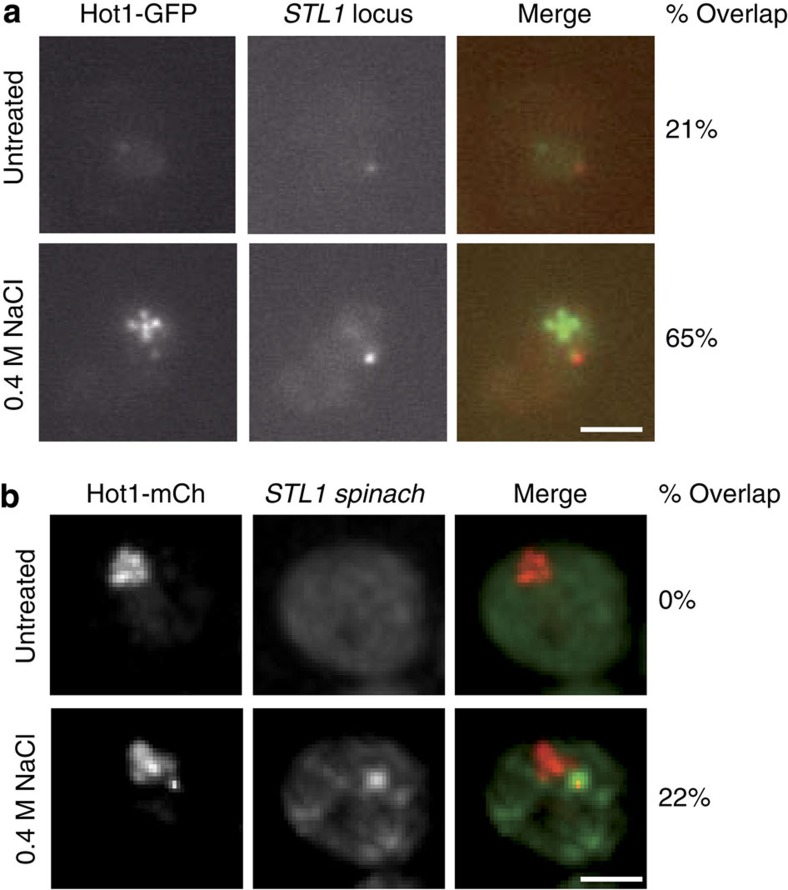
Co-localization of the transcription factor Hot1 with the *STL1* gene and transcripts. (**a**) Co-localization of the *STL1* gene and the osmotic stress-induced transcription factor Hot1. Cells were imaged from untreated (*n*=52) and 0.4 M NaCl-treated (*n*=105) cultures for Hot1–GFP and mCherry–LacI. (**b**) Co-localization of the *STL1* transcript and the osmotic stress-induced transcription factor Hot1. Cells expressing Hot1–mCherry and *STL1-*Spinach in the same field were imaged before and after a 5-min 0.4 M NaCl treatment (*n*=226). Scale bar, 2 μm.

In *S. cerevisiae*, the osmotic stress response is mediated by the Hog1 mitogen-activated protein kinase, which enters the nucleus and activates different transcription factors, such as Hot1, to initiate the transcriptional response^27^. During the stress, Hot1–GFP, and also Msn2– and Sko1–GFP, localization shifted to multiple distinct intra-nuclear foci (Supplementary Fig. 5). Before exposure to osmotic stress, the number of Hot1–GFP foci per nucleus ranged between 0 and 1. When cells were exposed to 0.4 M NaCl, the number of foci increased to 2–5, with 15% of cells exhibiting >5 foci per nuclei. The formation of these foci was partially affected in *hog1Δ* cells on osmotic stress, indicating that the Hog1 pathway is required but not sufficient to mediate this molecular event (Supplementary Fig. 5). The Hot1–GFP foci resembled the clustering of transcription events such as transcription factories reported in mammalian cells. To test this further, we localized the Hot1-responsive gene *STL1* and Hot1–GFP in untreated and induced cells. In untreated cells, 21% of the *STL1* loci co-localized with the Hot1–GFP foci. However, in 0.4 M NaCl-treated cells, the co-localization increased to ∼65% of the *STL1* loci with one of the Hot1–GFP foci (Fig. 5a). To analyse whether such foci correspond to transcription factories, Hot1–mCherry and *STL1 Spinach* transcripts were examined in induced live cells. Overlap between the transcription factor and the transcripts was observed in 22% of cells (*n*=226) within 5 min of osmotic stress and the transcription factor then dissociated from the *STL1* transcription area at later time points (Fig. 5b). Thus, nuclear dots where newly transcribed *STL1* RNAs accumulate probably correspond to the site of their transcription.

## Discussion

We show here that the use of RNA Spinach aptamer inserted between the coding region and the 3′-UTR of the gene of interest is an asset for mRNA imaging in live *S. cerevisiae* cells. We clearly demonstrate that the Spinach approach overcomes two limitations of the MS2- or PP7-based approaches, the constant fluorescent background due to the expression level of the GFP-tagged protein, as well as photobleaching effects and consecutive phototoxic effects. This technology can thus be used for a dense regime of image acquisition or alternatively long-term imaging at the appropriate space resolution, independently of the signal intensity. In contrast, changes in the signal intensity of MS2-tagged RNAs—for example, when 3′ rather than 5′ of gene open reading frame (ORF) are MS2 tagged—alter image acquisition performance^8^. In principle, the use of RNA Spinach aptamer thus represents an interesting alternative to the panel of existing techniques and opens new possibilities^28^. We specifically chose to tag mRNA with a unique Spinach aptamer rather than using an array of aptamer motives to avoid alterations of mRNA behaviour. However, it resulted in a very low signal, a challenging problem when compared with the MS2- or PP7-based approaches, where tandem GFPs are used to increase the signal. The very limited number of specific transcripts per yeast cell indeed led to a low signal-to noise-ratio that generally precludes accurate quantitative dynamic studies or requires higher light irradiation, with hazardous phototoxic effects. Here, to circumvent these major limitations, we propose a simple and adapted image-processing workflow as a robust alternative for the study of RNA biogenesis and trafficking events that occur in a timescale incompatible with an accurate analysis by FISH, or in a restricted volume that requires high-resolution approaches.

Fluorescence fluctuation spectroscopy has been used to provide quantitative information on MS2- or PP7-tagged mRNA dynamics in living cells expressing single-chain tandem dimers of the MS2 and PP7 coat proteins^29^. Moreover, the recent optimization of RNA aptamer/fluorophore complexes and their resulting yield of fluorescence^30^ should improve the signal-to-noise ratio and thus the sensitivity of the approach. Combining these recent developments—optimized aptamers and fluorescence fluctuation spectroscopy—could be an ideal approach for analysing single-molecule kinetics of diverse steps of mRNA nuclear biogenesis.

Our approach allows detection of nuclear mRNAs that transiently accumulate in a highly dynamic nuclear dot before their transport to the cytoplasm. We find that these nuclear RNAs correspond to newly transcribed RNAs as they co-localize with cognate gene loci and appropriate transcription factors, thus resembling mammalian transcription factories. In addition, the cell-to-cell variability of the Spinach signal in nuclear dots might reflect multiple transcripts being present at the transcription sites simultaneously, at least in some cells. The spatiotemporal dynamics we observe for both gene locus and corresponding nuclear transcripts confirm that osmostress-induced genes relocate towards the nuclear periphery to be transcribed. Our precise analysis of the *NUP60* deletion phenotype illustrates that a correct positioning of active gene at the nuclear periphery is not sufficient to promote an efficient transcription. This is in agreement with previous studies^21^,^31^. However, the role of Nup2 in defining the amplitude of *STL1*gene locus movements on activation suggests that integrity of the NPC is probably required for a correct spatial positioning of induced genes at the nuclear periphery. Activation of osmo-responsive genes is controlled by diverse transcription factors downstream of the Hog1 kinase such as Hot1 for *STL1* and Msn2/Msn4 for *CTT1* (ref. 32). Whether different transcription factories require different microenvironments at the nuclear periphery to efficiently associate with their target genes or to exert their full activity remains to be determined.

Importantly, we obtain improved localization precision using an adapted imaging workflow and reveal that active genes rarely co-localize with the nuclear envelope. In addition, the measured average distance of 300–350 nm between active gene loci (*STL1*, *CTT1* and *GAL10*) and nuclear periphery is not compatible with a stable and direct tethering of active genes to the NPC, as already suggested by analysis of galactose-inducible *GAL* genes^33^. Together we propose NPCs probably create dynamic nuclear microenvironments within the nuclear space that favour transcription activation and coordination with nuclear transport rather than a physical and static platform where transcription factories assemble.

## Methods

### Yeast strains and culture

Yeast strains used in this study are described in Supplementary Table 1. Cultures were maintained in YPD medium (1% yeast extract, 2% Bacto Peptone, 2% glucose) or in Synthetic Complete (SC) medium (0.67% yeast nitrogen base without amino acids, 2% glucose and 0.08% Complete Supplement Mixture dropout mixture). Cells were grown overnight at 30 °C in SC medium, reinoculated into fresh SC medium and grown at 30 °C for 4–6 h before microscopy. Osmotic shock was induced by adding NaCl to a 0.4 M final concentration. To induce *GAL1* expression, cells grown overnight in SC 2% raffinose were treated with a 2% final concentration of galactose.

### Spinach tagging

Spinach 24–2 Min (Spinach^11^) DNA fragments generated by annealing complementary synthetic oligonucleotides were inserted into EcoR V site of pUG27 vector (His5 marker), to obtain pUG27-Spinach plasmid (template). A PCR product was generated using Gene-Spinach F and R primers. Primer Gene-Spinach F contains a sequence of 40 nucleotides corresponding to the 3′-end of the ORF (including the stop codon) followed by a 20-nucleotide sequence corresponding to the template plasmid. Gene-Spinach R contains the reverse complement of the sequence corresponding to the first 40 nucleotides of the 3′-UTR immediately after the stop codon followed by a 20-nucleotide sequence corresponding to the template plasmid. This PCR product was transformed into yeast and was integrated by homologous recombination into the gene, between the coding region and the 3′-UTR. Yeast cells were then transformed with the plasmid pSH47 (Euroscarf, URA marker), to allow expression of the CRE recombinase from a galactose-inducible promoter, thus resulting in the excision of the His5+ marker. Medium containing 5-FOA was then used to eliminate the pSH47 plasmid. After His5+ excision, 67 nucleotides remain between stop codon and Spinach sequence. Oligonucleotides used in this study are described in Supplementary Table 2.

### RNA isolation and amplification

Total RNA isolation was performed by the hot acid phenol method. Complementary DNA from total RNAs were obtained by retro-transcription with random oligonucleotides (Roche) using the SuperScript II reverse transcriptase (Invitrogen). Real-time qPCR was then performed using the SYBR Green mix (Roche) and the Light Cycler 480 system (Roche) with gene-specific primers described in Supplementary Table 3.

### Flow cytometry

Yeast cultures grown to late log phase were diluted and grown for at least 15 h to an OD_600_ of 0.5. The cultures were then treated with 0.4 M NaCl for the various time points and then harvested by diluting into 10 mM Tris, 1 mM EDTA pH 8.0, plus 1 μg ml^−1^ cyclohexamide. The STL1–GFP intensity was immediately quantified using a Guava easyCyte Flow cytometer. SSC and FSC parameters were used to collect 20 000 cells that excluded doublets and small debris^20^. Data were graphed using FlowJo software.

### FISH experiments

FISH was performed as previously described^34^. 5′-Cy3-end-labelled 60-bp oligonucleotides were used to cover the ORF region of the *STL1* gene and a mix of four probes for the *ASH1* gene^21^. The wt and *nup60Δ* cells were grown to *A*_600_=0.5. Cells were treated with 0.4 M NaCl for 5, 10, 15 or 20 min and fixed with 4% formaldehyde. The nucleus was stained with 4,6-diamidino-2-phenylindole and cells were mounted in Mowiol. Pictures were taken using a Leica DM5000B fluorescence microscope with a × 100/1.4 numerical aperture (NA) HCX PL APO objective and a Z-piezo controller.

### Microscopy

Imaging of transcription factor foci and quantification of Hot1 foci were performed in untreated cells and after a 5-min shift to 0.4 M NaCl. Cultures for imaging were diluted from saturated overnight starter cultures to an OD_600_ of 0.05 and then grown at 30 °C for 5 h to an OD_600_ of 0.4–0.6. Images were acquired with a DeltaVision microscope system (Applied Precision, IX70; Olympus) using a × 100/1.40 NA UPlanSApo objective and Photometrics CoolSnap HQ2 camera. Images were processed with softWoRx imaging software and DeltaVision's constrained 3D deconvolution method. Further linear adjustments were made for brightness and contrast in ImageJ or Adobe Photoshop CS6.

GFP–LacI/*STL1*-LacO images analysed for overlap with the nuclear periphery marked by DsRed-HDEL or with Hot1–GFP (Supplementary Fig. 4 and Fig. 5) were acquired with a standard microscope (BX50; Olympus) equipped with a motorized stage (Model 999000, Ludl), UPlanF1 × 100/1.30 NA oil-immersion objective and digital CCD (charge-coupled device) camera (Orca-R2; Hamamatsu). Image processing was performed in NIS-Elements (Nikon), ImageJ (NIH) and Adobe Photoshop CS6. GFP–LacI/*STL1*-LacO images analysed for overlap with the nuclear periphery marked by anti-Nup53 (Abcam Ab 11692; used at the 1/250 dilution) were acquired in 3D SIM mode with an N-SIM microscope (Nikon).

### Live-cell imaging

Before acquisition, cells were plated on a glass-bottom dish (IBIDI μDish 35 mm high glass bottom) coated with 1 mg ml^−1^ concanavalin A (Sigma), for at least 10 min. Control of temperature was set to 30 °C. For spinach tagging, SC media was buffered to pH 6, to avoid any precipitation of DFHBI (Lucerna Technologies). Moreover, to prevent nonspecific increase of fluorescence signal on induction, 100 μM DFHBI was included to the media before and after activation.

*Dynamics of STL1-Spinach activation*. Spinning-disk confocal microscopy was carried out with a Yokogawa CSU-X1 spinning disc head on a Nikon Eclipse Ti inverted microscope equipped with an electron multiplying CCD camera (Evolve 512, Photometrics), a NanoScanZ piezo focusing stage (Prior Scientific), and a motorized scanning stage (Marzhauser) and a Nikon S Fluor × 100/1.4 NA objective. For spatiotemporal study, Z-stack of 11 planes spaced by 0.3 μm were acquired every 5 s for 10 min, starting 2 min after induction with 0.4 M NaCl in the case of *STL1 Spinach*. The osmotic shock was performed directly under the microscope. For more dynamical studies, a single plane (or two planes) was acquired each second for 10 min. This microscope was driven with Metamorph and images shown were processed with Image J and assembled with Adobe Photoshop CS6.

*Dynamics of STL1 gene locus localization*. Confocal images were taken on a fully motorized inverted microscope (Eclipse Ti-E; Nikon) controlled with MetaMorph software 7.7.8 and equipped with the Perfect Focus System (Nikon) to maintain the focus, a × 100/1.45 NA Plan Apochromat oil-immersion objective, a spinning-disk confocal unit (CSUX1; Yokogawa Corporation of America), a CCD camera (CoolSNAP HQ2, Photometrics) and a laser bench (Roper Scientific SAS) with 491- and 561-nm diode lasers (100 mW each; Cobolt). To study the localization of *STL1* gene locus on induction, Z-stack images of 14 planes spaced by 0.3 μm were acquired before induction and 10 min after activation.

### *mRNA Spinach* quantitative image analysis

To track the activation of mRNAs both within and outside the nucleus for a given yeast cell, a robust 3D detection and tracking algorithm was designed and implemented using Matlab and the CImg C++ image library. The nuclear envelope and the mRNA spots were detected, in the 3D stack, using DoG filters and a threshold defined by a *P*-value (with different *σ*-and *P*-value for the nucleus and mRNA spots).

After identifying the nuclear envelopes within a given 3D stack using the DoG detection algorithm, every nuclear volume (within the 3D stack before activation) was labelled separately. The Euclidean distance between the centroid of a given nuclear volume was then computed with the centroids of all the nuclei volumes in the corresponding time of acquisition. The one with the minimum distance and less than a given threshold was considered to be identical and was assigned the same label. The process was repeated over consecutive time points, to follow the nucleus over the total time of operation. If the minimum Euclidean distance for a given nucleus exceeded the threshold value, the algorithm did not detect any nucleus for that particular time point (this generally corresponds to photobleaching). Once the nucleus was labelled, the mRNA spots within the nucleus and the corresponding cytoplasmic volume were labelled in accordance with the nuclear label, thereby enabling us to follow the movement of the *STL1* mRNA within the 3D stack, over the given time of operation.

The fluorescent intensity values corresponding to the *STL1* mRNA spots (detected as masks using DoG filters), inside the nuclear and the cytoplasmic volume, were then plotted over time as intensity maps (see Fig. 2d and Supplementary Fig. 3; the change in fluorescent intensity of the *STL1* mRNA for a given nucleus and corresponding cytoplasm, with dark blue indicating no detection of *STL1* mRNA and red indicating its maximum fluorescent intensity at a given time point). The absolute fluorescence intensity in the nucleus and cytoplasm can be represented with the same scale for the complete cell population (unnormalized) or alternatively normalized for each cell (normalized). From the normalized intensity maps, the activation times for *STL1* mRNA were calculated by non-zero mean thresholding their fluorescent intensity values (within a given nucleus and its cytoplasm) over the time of operation and then locating the position of the first value from the thresholded intensity map. The computation was done for a total of 235 cells and the cumulative distribution function was plotted (see Fig. 2e) to compare the time of detection of *STL1* mRNA in the nucleus and in the cytoplasm.

### Gene gating quantitative image analysis

To improve the spatial resolution of the acquired spinning-disk microscopy images, a dedicated software *ndsafir*^16^ was used to denoise the images followed by Gold–Meinel deconvolution^23^ of the images using a measured PSF of the microscope. The 3D nuclear envelope and the *STL1* gene were then identified as binary masks using the same detection technique used in the mRNA spinach analysis. Labelling was done accordingly between the initial and final time point of the acquisition, to match the corresponding nuclei and their *STL1* gene.

The Euclidean minimum distance (denoted as *d* between set of *N*_p_ pixels of the periphery pixels of the nuclear mask of coordinates {(*x*^*i*^_nuc_, *y*^*i*^_nuc_, *z*^*i*^_nuc_), *i=*1:*N*_p_}) and the centroid of the *STL1* gene (detected as spots by DoG filters) of coordinates (*x*_gene_, *y*_gene_, *z*_gene_) was considered taking into account the anisotropy of the voxel (64 × 64 × 300 nm) of the acquired volume. This computation was done for all nuclei with genes detected in their initial and final time points. By considering the difference *d*_diff_ of this distance before and after induction, the displacement of the *STL1* gene was estimated as either towards the nuclear envelop (negative value) or away from it (positive value).

The *d*_diff_ over a total of 95 yeast nuclei (considered to be spherical) was computed both for the wt and deletion mutants. The distribution of *d*_diff_ values against the percentage of cells was generated as histogram plots (see Fig. 3b), giving a distribution of the percentage of cells observing shift in the position of the *STL1* gene, on activation, over a given range.

## Additional information

**How to cite this article:** Guet, D. *et al.* Combining Spinach-tagged RNA and gene localization to image gene expression in live yeast. *Nat. Commun.* 6:8882 doi: 10.1038/ncomms9882 (2015).

## Supplementary Material

Supplementary Information Supplementary Figures 1-5 and Supplementary Tables 1-3.

Supplementary Movie 1 Fast time-lapse movie of STL1 activation. STL1 Spinach signal induced upon treatment with 0.4M NaCl was recorded after the induction to avoid side effects of cell shrinkage. Images were acquired by spinning-disk microscopy on a single plane with one frame/second for 10 min. Nuclear transcripts appeared as a nuclear dot. The nuclear periphery was visualized with Nup159-mCherry. 3 fold rescale of the original crop.

Supplementary Movie 2 Induction of STL1 transcript in WT. STL1 Spinach signal induced upon treatment with 0.4M NaCl in cells expressing Nup159-mCherry was recorded 2 min after induction with one Z stack of 11 frames/5 s. Each Z-stack was first denoised using nd-Safir. The movie corresponds to a maximum projection of the 11 frames of the denoised stacks.

Supplementary Movie 3 Induction of STL1 transcript in nup60?? strain. STL1 Spinach signal induced upon treatment with 0.4M NaCl in cells expressing Nup159-mCherry was recorded 2 min after induction with one Z stack of 14 frames/20 s. Each Z-stack was first denoised using nd-Safir. The movie corresponds to a maximum projection of 6 frames of the denoised stacks.

## Figures and Tables

**Table 1:** Quantitative analysis of *STL1* positioning.

**(nm)**	**wt**	**nup60Δ**	**nup2Δ**
Mean distance before activation	500	450	650***
			
Mean distance after activation			
Towards the periphery	350	300	475**
Towards the centre	550	550	750***
			
Mean shift after activation			
Towards the periphery	200	200	250*
Towards the centre	150	200	225**

Distance between the *STL1* gene locus before and after activation, as well as the mean shift after activation have been measured from cells analysed in Fig. 4c (*n*=95 wt cells, *n*=95 for *nup60Δ* cells and *n*=91 for *nup2Δ* cells). Results from mutant cells have been compared with wt cells using a Student's *t*-test, ***P<0.001, **0.001<*P*<0.01, *0.01<*P*<0.05.
