# Supplementary material for: Combining Spinach-tagged RNA and gene localization to image gene expression in live yeast
Source: Nat Commun. 2015 Nov 19;6:8882. doi: 10.1038/ncomms9882 (PMC4673486; doi:10.1038/ncomms9882)
Supplement: Supplementary Information — Supplementary Figures 1-5 and Supplementary Tables 1-3. [file ncomms9882-s1.pdf]

Supplementary Figures

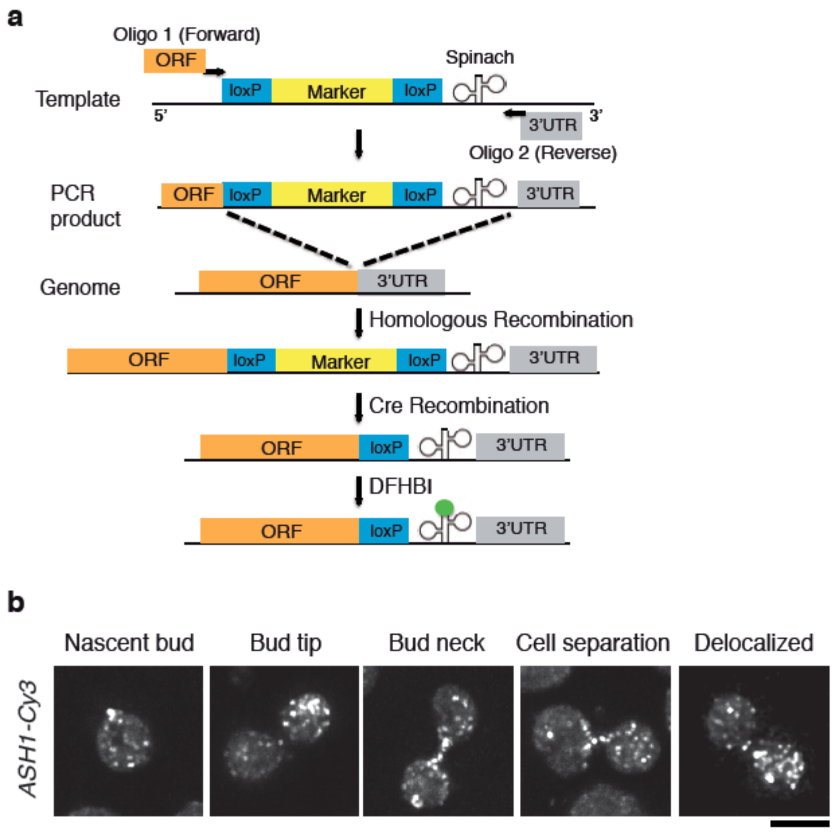

Supplementary Figure 1: (a) Schematic representation of the strategy to express endogenous Spinach-tagged mRNAs. Spinach RNA sequence was introduced in a template plasmid downstream of the LoxP-flanked selection marker Sp his5+. DNA encoding marker and Spinach were amplified by PCR using primers corresponding to the genome sequence of interest. To prevent interference with the 3'end processing, Spinach is inserted between the coding region and the 3'-untranslated region of the gene of interest. The PCR product is then introduced in the yeast genome by homologous recombination. After a positive selection using the marker, expression of Cre recombinase allows removal of the marker sequence in order to reduce the length of the RNA tag to the length of Spinach, allowing expression of the chimeric and fluorescent mRNA in presence of DFHBI. **(b) Localization of *ASH1* mRNAs analyzed by FISH.** Examples of ASH1 transcripts localization at different stages of the cell cycle. Corresponding quantifications are shown in Figure 1. Bar 3 µm.

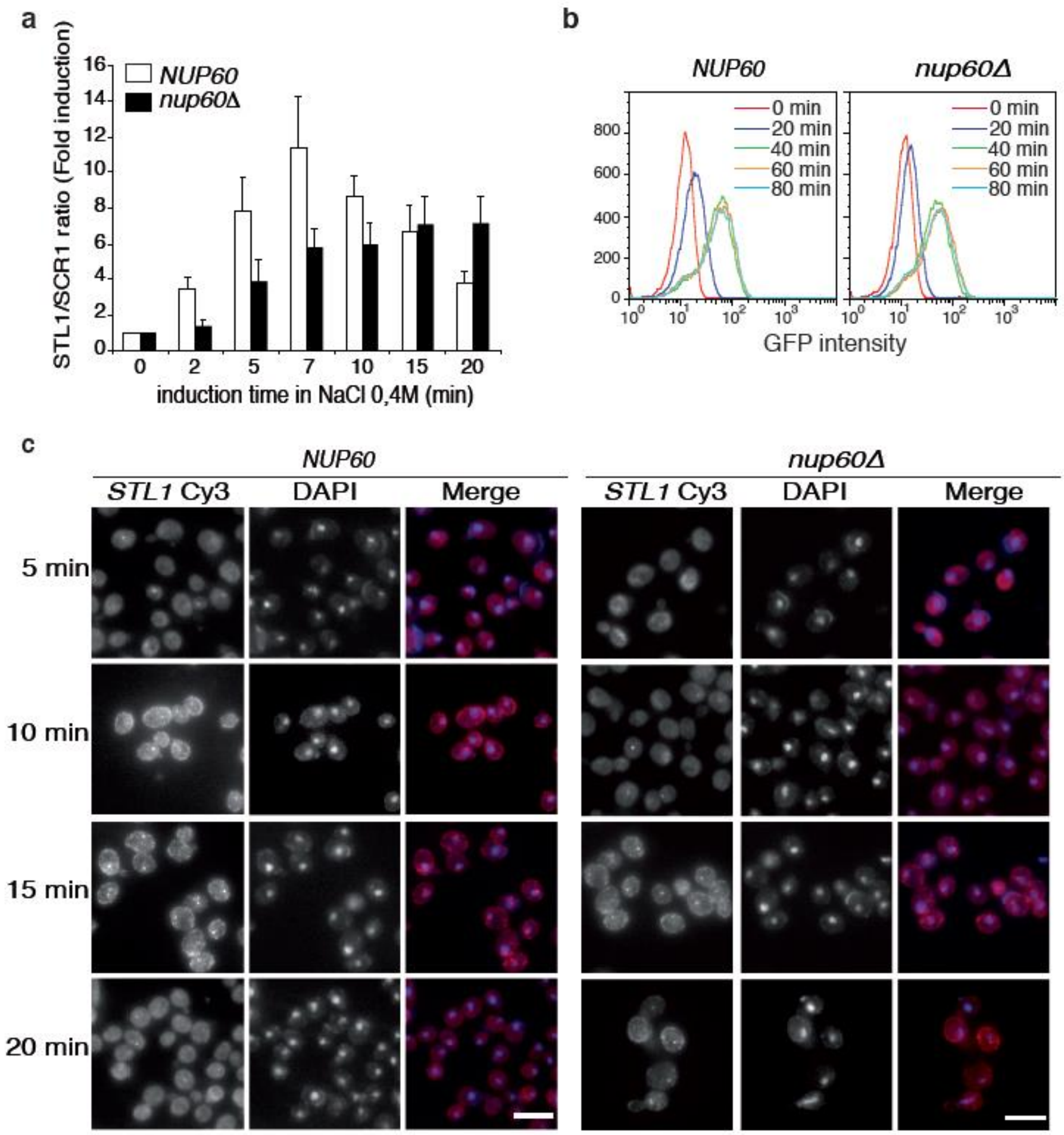

Supplementary Figure 2: Analysis of *STL1* transcripts by usual approaches (a) Expression of *STL1-Spinach* transcripts. Expression of *STL1* transcripts was analyzed in 6 independent experiments by RT-qPCR in wt and *nup60Δ* mutant cells, normalized to the expression of SCR1 transcript and non induced condition. Errors bars indicate standard deviations. **(b) Expression of STL1-GFP.** Expression of GFP-tagged STL1 protein was analyzed by flow cytometry in wt and *nup60Δ* mutant cells at different times after a 0.4M NaCl stress. **(c) Localization of STL1 transcripts by FISH.** STL1 transcripts were localized by FISH using specific probes in wt and *nup60Δ* mutant cells at different time points after a 0.4M NaCl stress. Bar, 10 μm

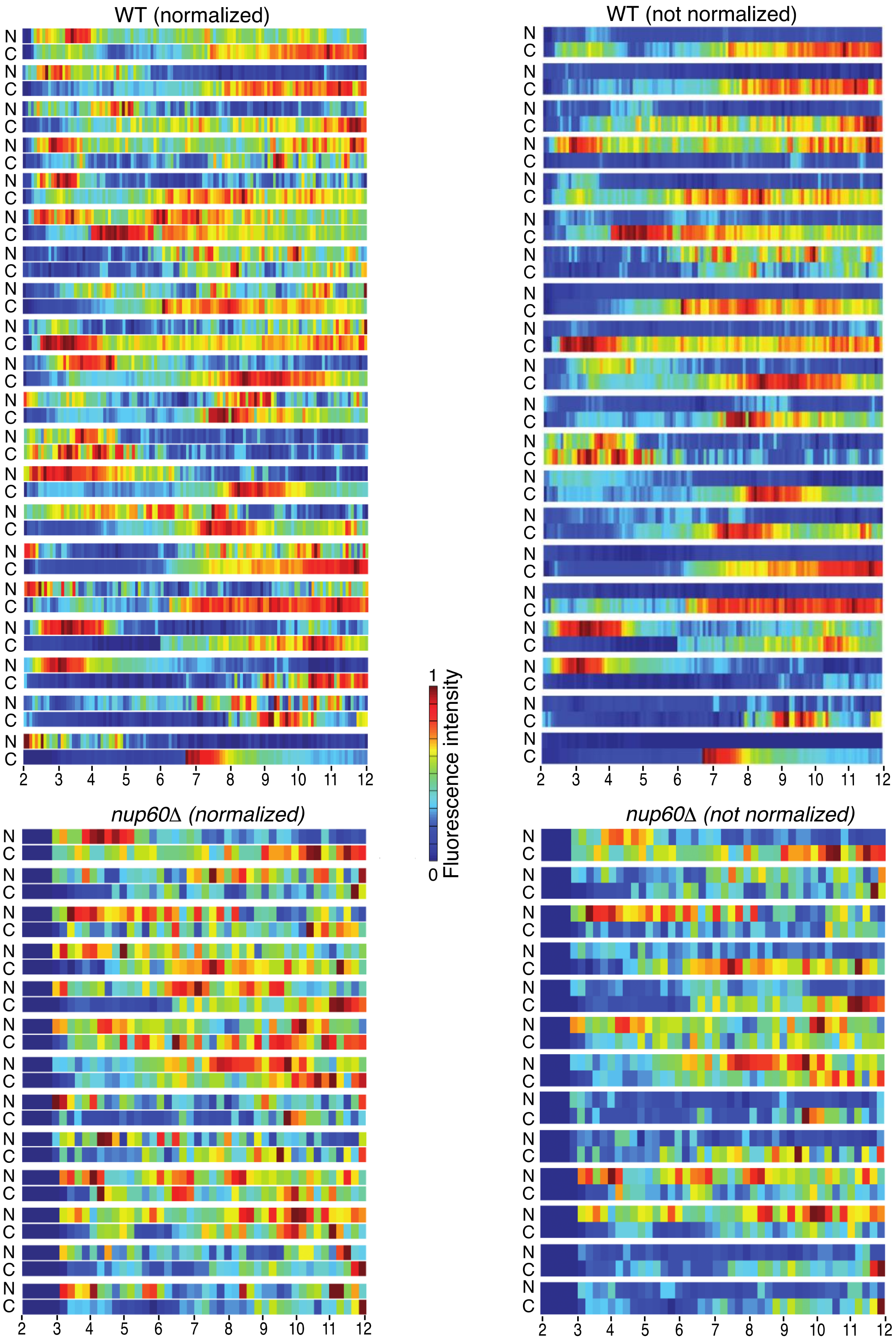

Supplementary Figure 3: Gallery of *STL1 Spinach* fluorescence intensity heat maps. The fluorescence intensity corresponding to *STL1 Spinach* mRNAs was measured over time both in the nucleus (N) and in the cytoplasm (C) as described in Figure 2c, and represented using heat maps corresponding to each cell. The absolute fluorescence intensity in nucleus and cytoplasm

can be represented with the same scale for the complete cell population in order to compare each single cell within the population (unnormalized) or alternatively normalized for each cell to further analyzed the fluorescence dynamics in each single cell (normalized). Examples of heat maps obtained for WT (upper panels) and *nup60Δ* (lower panels) mutant cells are represented.

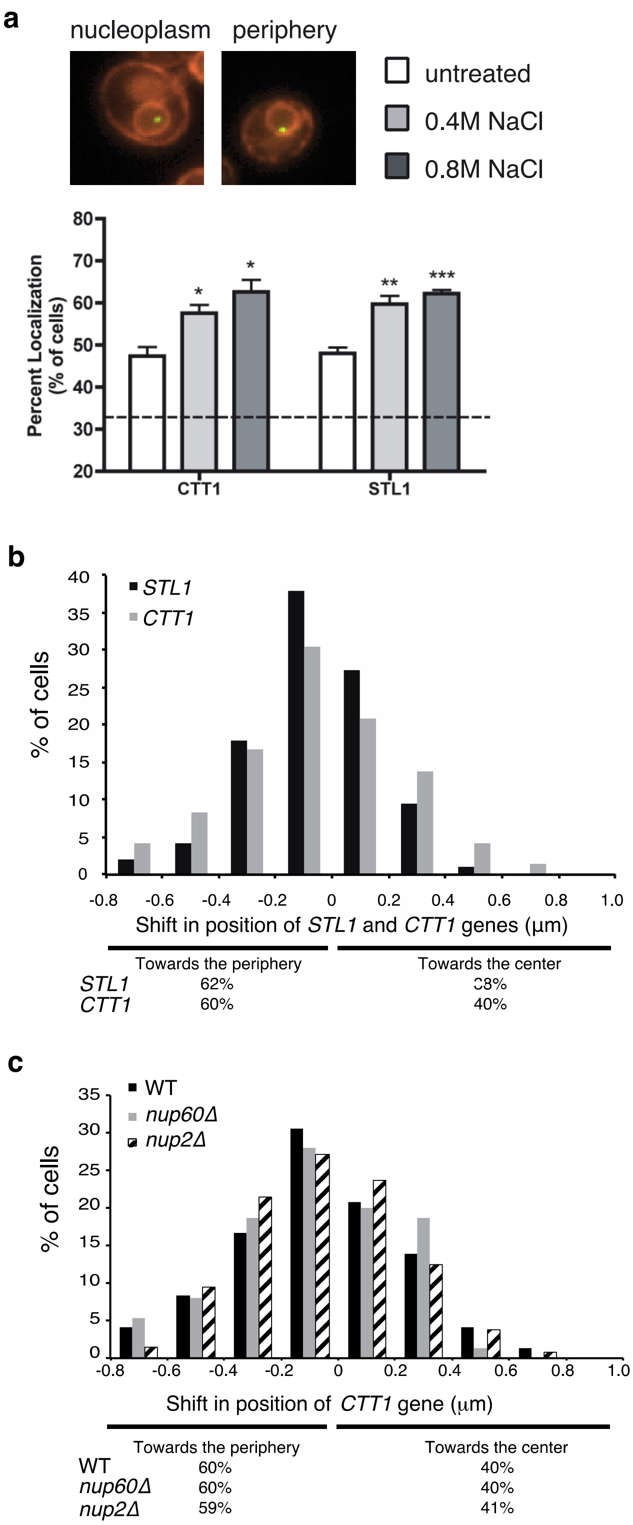

Supplementary Figure 4: Localization of *CTT1* gene locus. (a) Peripheral positioning of osmotically-induced *STL1* and *CTT1* gene loci. Overlapping GFP-LacI/STL1-LacO and DsRed-HDEL signals were quantified in untreated cells and after treatment with 10 minutes of 0.4 M NaCl and 30 min of 0.8 M NaCl. Z-stacks were acquired with wide-field epifluorescent

microscopy and analyzed using the "3 zones" quantification in 2D. n=50 with three experimental replicates. Errors bars indicate standard deviations. *p < 0.05, **p < 0.01, *** p < 0.001. **(b) *STL1* and *CTT1* gene positioning upon activation in WT cells**. The shift in position of the gene was calculated in 3D on n=95 cells for *STL1* and n=72 for *CTT1* recorded as in Figure 4. A negative shift corresponds to gene moving close to the nuclear periphery whereas a positive shift describes movement towards the center of nucleus. **(c) *CTT1* gene positioning upon *NUP60* and *NUP2* deletion**. The shift in position of the gene was calculated in 3D on n=72 wt cells, n=75 for *nup60Δ* cells and n=266 for *nup2Δ* cells recorded as in Figure 4. Total % of cells presenting a shift of *STL1* gene locus towards the periphery or the center is indicated for the different loci and strains.

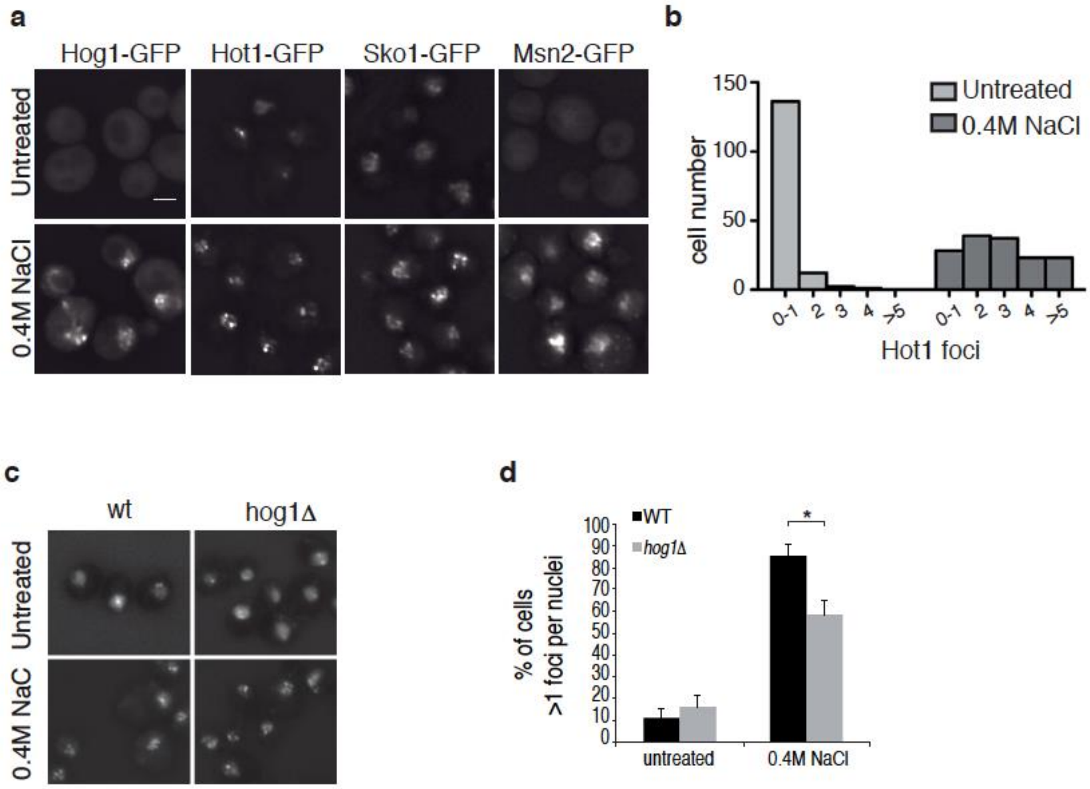

Supplementary Figure 5: Subcellular localization of osmostress-induced transcription factors (a) C-terminal GFP-fusions of Hog1, Hot1, Sko1, and Msn2 were visualized by live cell microscopy in YPD and after 5 minutes of 0.4M NaCl stress. **(b)** Quantification for Hot1-GFP foci in wildtype cells untreated and under hyperosmotic stress (0.4M NaCl). (**c**) Hot1-GFP localization by live cell microscopy in strains deleted for *HOG1*. **(d)** Quantification for Hot1-GFP foci in *hog1Δ* mutant strain relative to wildtype untreated and under hyperosmotic stress (0.4M NaCl). Bars represent standard error, **p<0.01, relative to wildtype.

Supplementary Tables

Supplementary Table 1 : Strains used in this study

Name	Genotype	Reference
BY4741 WT	Matα; his3Δ1; leu2 Δ 0; met15 Δ 0; ura3 Δ 0	Euroscarf
BY4741 *nup60Δ*	isogenic to WT; *NUP60*::kanMX4	This study
STL1-Spinach	isogenic to WT; *STL1*-SpinachPlus	This study
GAL1-Spinach	isogenic to WT; *GAL1*-SpinachPlus	This study
STL1-Spinach Nup159-mCherry	isogenic to WT ; *STL1*-SpinachPlus ; *NUP159*-mCherry::kanMX4	This study
GAL1-Spinach Nup159-mCh	isogenic to WT ; *GAL1*-SpinachPlus ; *NUP159*-mCherry::kanMX4	This study
ASH1-Spinach Nup159-mCh	isogenic to WT ; *ASH1*-SpinachPlus ; *NUP159*-mCherry::kanMX4	This study
ASH1-Spinach Nup159-mCh *nup60Δ*	isogenic to WT ; *ASH1*-SpinachPlus ; *NUP159*-mCherry::kanMX4, NUP60::HPH	This study
STL1-Spinach Nup159-mCherry *nup60Δ*	isogenic to WT ; *STL1*-SpinachPlus ; *NUP159*-mCherry::HPH ; NUP60::kanMX4	This study
STL1-Spinach Hot1-mCherry	isogenic to WT; *STL1*-SpinachPlus ; *HOT1*-mCherry:KAN his3	This study
SWY4715	isogenic to WT ; *STL1*-LacO::URA3, *NIC96*-mCherry::HPH, his3::LacI-GFP	
SWY4715 *nup60Δ*	isogenic to WT ; *STL1*-LacO::URA3, *NIC96*-mCherry::HPH, his3::LacI-GFP, *NUP60*::kanMX4	This study
SWY4715 *nup2Δ*	isogenic to WT ; *STL1*-LacO::URA3, *NIC96*-mCherry::HPH, his3::LacI-GFP, *NUP2*::kanMX4	This study
SWY4714	isogenic to WT ; *CTT1*-LacO::URA3, *NIC96*-mCherry::HPH, his3::LacI-GFP	
SWY4714 *nup60Δ*	isogenic to WT ; *CTT1*-LacO::URA3, *NIC96*-mCherry::HPH, his3::LacI-GFP, NUP60::kanMX4	This study
SWY4714 *nup2Δ*	isogenic to WT ; *CTT1*-LacO::URA3, *NIC96*-mCherry::HPH, his3::LacI-GFP, *NUP2*::kanMX4	This study

Supplementary Table 2 : Oligonucleotides used in this study

**Name**	**Sequence**
**Spinach EcoR5 F**	GATATCGCCCGGATAGCTCAGTCGGTAG
**Spinach EcoR5 R**	GATATCTGGCGCCCGAACAGGGACTTG
**Spinach blunt F**	GCCCGGATAGCTCAGTCGGTAG
**Spinach blunt R**	TGGCGCCCGAACAGGGACTTG
**pUG27 nd 1674 R**	GGATAACCGTATTACCGCCTTTG
**Spinach 37 F**	CAACTGAATGAAATGGTGAAGGAC
**Spinach 103 R**	CGACTAGTTACGGAGCTCACACTC
**PUG27 962 F**	CACTTACTATATTCCTTTTCGGTAGC
**PUG27 1870 R**	GGAGAGCGCACGAGGGAGCTTCCAG
**STL1-SpinachPlus F**	TGAAGATACAGTGAACGATAAAGCAAATTTTGAGGGTTG AACGCTGCAGGTCGACAACCC
**STL1-SpinachPlus**	TAAGTAAATTACAAAATATGATTTGTGAGTTGTGTGTGAA

**R**	GCATAGGCCACTAGTGGATC
**GAL1-SpinachPlus F**	CTCTAAACCAGCATTGGGCAGCTGTCTATATGAATTATAA AACGCTGCAGGTCGACAACCC
**GAL1-SpinachPlus R**	TGAGAAGTTGTTCTGAACAAAGTAAAAAAAAGAAGTATA CGCATAGGCCACTAGTGGATC
**ASH1-SpinachPlus F**	CTTATTTTGTAATTACATACTGAGACAGTAGAGAATTGAA CGCTGCAGGTCGACAACCC
**ASH1-SpinachPlus R**	ATGTCTCTTATTAGTTGAAAGAGATTCAGTTATCCATGTA GCATAGGCCACTAGTGGATC
**Nup159-mCherry F**	GAATACGAAAAAGCAAATTGGTGATTTCTTCAAAAATTTG AACATGGCAAAACGTACGCTGCAGGTCGAC
**Nup159-mCherry R**	GAATTTATTATTAACGGCACTAACAACGTACATATAGCTAA ATATCACTAATCGATGAATTCGAGCTCG
**Hot1-mCherry F**	AAGAAAAGAACCATTGGCTGGTTGCAAGAGAGCCTTGCTG GAATACGTACGCTGCAGGTCGAC
**Hot1-mCherry R**	CCTTCCTTCCTATGATTGTAAACGATTATTTACTATCGTACG TGCATCGATGAATTCGAGCTCG
**Nup60-deletion F**	ATCAAATAAGCACCGCAAGATATCCTAAAATCGACATCCA CGGATCCCCGGGTTAATTAA
**Nup60-deletion R**	GGGCTATACGGTAATTATGTCACGGCTAAAATTTTCATTAG AATTCGAGCTCGTTTAAAC
**Nup2-deletion F**	CTCAAAAAAATCATTAACGAGCGGATCCCCGGGTTAATTAA
**Nup2-deletion R**	TGTTAACTGTATTTACTCTTAGAATTCGAGCTCGTTTAAAC

Supplementary Table 3 : Oligonucleotides used for RT-qPCR

**Oligonucleotides**	**Sequence 5'-3'**
PMA1 5' forward	TCAGCTCATCAGCCAACTCAAG
PMA1 5' reverse	CGTCGACACCGTGATTAGATTG
SCR1 forward	GTAATGGCTTTCTGG
SCR1 reverse	GTGCGGAATAGAGAACTATCC
GAL1 forward	CAGAGGGCTAAGCATGTGTATTCT
GAL1 reverse	GTCAATCTCTGGACAAGAACATTC
STL1 5' forward	TTACGGGTAAGAAGTTGAGG
STL1 5' reverse	TGTCTGTCATGCTCGCCATT
